# Supplementary material for: Probing coherent quantum thermodynamics using a trapped ion
Source: Nat Commun. 2024 Aug 14;15:6974. doi: 10.1038/s41467-024-51263-3 (PMC11324868; doi:10.1038/s41467-024-51263-3)
Supplement: Supplementary file 1 — Supplementary Information [file 41467_2024_51263_MOESM1_ESM.pdf]

Supplementary Notes: Probing coherent quantum thermodynamics using a trapped ion

O. Onishchenko*,[1] G. Guarnieri*,[2,3] P. Rosillo-Rodes,[4] D. Pijn,[1] J. Hilder,[1]
U. G. Poschinger,[1] M. Perarnau-Llobet,[5] J. Eisert,[3] and F. Schmidt-Kaler[1]

[1]*QUANTUM, Institut für Physik, Universität Mainz, Staudingerweg 7, 55128 Mainz, Germany**
[2]*Department of Physics and INFN - Sezione di Pavia, University of Pavia, Via Bassi 6, 27100, Pavia, Italy*
[3]*Dahlem Center for Complex Quantum Systems, Freie Universität Berlin, 14195 Berlin, Germany**
[4]*Institute for Cross-Disciplinary Physics and Complex Systems,*
Campus Universitat de les Illes Balears, E-07122, Palma, Spain
[5]*Department of Applied Physics, University of Geneva, 1211 Geneva, Switzerland*

This Supplementary Information contains additional details about the the derivation of the evaluation of the work distribution, the data analysis and the experimental implementation of the protocol operations. In particular, Supplementary Note 1 provides further insight and derivation of Eq. (9) of the main text, while Supplementary Note 2 contains a detailed discussion of all relevant error sources affecting the data shown in Figs. 2 and 3 of the main manuscript. Finally Supplementary Note 3 includes additional details about the experimental setup and implementation.

I. SUPPLEMENTARY NOTE 1: ANALYTIC EXPRESSIONS FOR THE CUMULANTS OF THE WORK DISTRIBUTION

In this Supplementary Note, we provide explicit expressions for the first and second cumulants of the work distribution distribution, i.e., its mean $\langle W\rangle$ and its variance $\mathrm{Var}(W)$, for the proposed coherent protocol describing the driving. At the end of this Section, we will also describe in detail the related expressions for the incoherent protocol that is calculated for comparison in Fig. 2 of the main text. Let us therefore start from the general expression of the work probability distribution Eq. (6) and apply it to the fully coherent protocol detailed in the main text and implemented experimentally. Simple calculations then lead to the following step work probabilities to measure positive or negative work within the TPM protocol:

$$P(w_j=+1) = (1-p)\,p_f, \tag{1}$$
$$P(w_j=-1) = p\,p_f, \tag{2}$$
$$P(w_j=0) = 1-P(w_j=+1)-P(w_j=-1). \tag{3}$$

Here, p denotes the thermal excited state population from Eq. (3) of the main text and p_f denotes the flip probability induced by the qubit rotation. We remark that these expressions can be straightforwardly obtained from the general expression Eq. (6). In the case of the coherent protocol describing the driving $\hat{H}^i_{\text{coh}} = -\hbar\omega_q\hat{\sigma_z}/2$ to $\hat{H}^f_{\text{coh}} = \hbar\omega_q\hat{\sigma}_y/2$, the ideal flip probability for the case of N subdivisions is given by

$$p_f^{(\text{ideal})} = \sin^2\left(\frac{\pi}{4N}\right). \tag{4}$$

Using this probability, one can compute the first two cumulants of the total work accumulated, which read

$$\langle W\rangle = N\,(1-2p)\sin^2\left(\frac{\pi}{4N}\right), \tag{5}$$
$$\mathrm{Var}(W) = N\sin^2\left(\frac{\pi}{4N}\right)\left(1-\sin^2\left(\frac{\pi}{4N}\right)(1-2p)^2\right). \tag{6}$$

Expanding these expressions at leading order in the number of steps corresponds to the slow-driving regime,

$$\langle W\rangle = \tanh\left(\frac{\beta}{2}\right)\frac{\pi^2}{4N} + \mathcal{O}(1/N^2), \tag{7}$$

$$\mathrm{Var}(W) = \frac{\pi^2}{4N} + \mathcal{O}(1/N^2). \tag{8}$$

---

* These authors contributed equally to this work..

This implies that the quantum correction $\mathcal{Q}$, in the protocol we experimentally implement, has the analytical expression

$$\begin{aligned}\mathcal{Q} &= \frac{\beta}{2}\mathrm{Var}(W) - \langle W\rangle \\ &= N\sin^2\left(\frac{\pi}{4N}\right)\left[\frac{\beta}{2}\left(1-\sin^2\left(\frac{\pi}{4N}\right)(1-2p)^2\right)-(1-2p)\right] \\ &= \frac{\pi^2}{4N}\left[\frac{\beta}{2}-\tanh\left(\frac{\beta}{2}\right)\right]+\mathcal{O}(1/N^2),\end{aligned} \tag{9}$$

where we use the relation $1-2p=\tanh\beta/2$ (we remind that we set $\hbar\omega_0=1$) . First of all, this expression clearly shows that $\mathcal{Q}\geq 0$ for all N, since

$$\frac{\beta}{2}\geq\tanh\left(\frac{\beta}{2}\right) \tag{10}$$

for all $\beta>0$. Furthermore, it highlights that the quantum correction vanishes in the high-temperature limit $\beta\to 0$ as $\tanh(x)\approx x+\mathcal{O}(x^2)$, thus leading to the standard fluctuation-dissipation relation (see Eq. (1) of the main text).

A. Incoherent protocol

We conclude this Supplementary Note by providing further details about the fully incoherent protocol that is considered in the main text in relation to the analysis of Fig. 2. The latter consists of a dynamical change of the qubit energy gap according to

$$\hat{H}(\omega_t)=\frac{\hbar\omega_q(t)}{2}\sigma_z, \tag{11}$$

from an initial value $\omega_q^{(i)}$ to a final value $\omega_q^{(f)}$ in N discrete equal steps of $\delta\omega=\frac{\omega_q^{(f)}-\omega_q^{(i)}}{N}$. For the jth step, we therefore have $\hat{H}(\omega_q^{(j)})$ with $\omega_q^{(j)}\doteq\omega_q^{(i)}+j\delta\omega$. Notice that these protocols are called fully incoherent since they generate no quantum friction due to the fact that $\left[\hat{H}(\omega_q^{(j)}),\hat{H}(\omega_q^{(j+1)})\right]=0, \forall j=1,\ldots,N$.

Starting once again from the general expression of the work statistics given by the TPM approach Eq. (6) of the main text, we have that the step work probabilities are given in this case by

$$P(w_j=\delta\omega)=p_j, \tag{12}$$

$$P(w_j=0)=1-p_j, \tag{13}$$

where we have defined

$$p_j=\frac{1}{1+e^{\beta\omega_q^{(j)}}} \tag{14}$$

as the excited state probability at the j-th step (after thermalisation). The total average work then becomes

$$\langle W\rangle=\delta\omega\sum_j p_j, \tag{15}$$

whereas the variance reads

$$\mathrm{Var}(W)=(\delta w)^2\sum_j p_j(1-p_j). \tag{16}$$

Note that in the incoherent protocol that the free energy difference ΔF, while vanishing in the coherent protocol, is non-zero. For each fixed pair $(\omega_q^{(i)},\omega_q^{(f)})$ of initial/final states, it reads

$$\Delta F=\frac{-1}{\beta}\ln\frac{Z_f}{Z_i}, \tag{17}$$

where $Z_{i,f}=\mathrm{Tr}\left[e^{-\beta\hat{H}(\omega_q^{(i,f)})}\right]$ denotes the partition function. From these expressions, one finds that the correction $\mathcal{Q}=\frac{\beta}{2}\mathrm{Var}(W)-(\langle W\rangle-\Delta F)$ decays as $\mathcal{O}(1/N^2)$ for any such incoherent process (see Fig. 2 in the main text and also [1]).

Finally, in order to compare coherent to incoherent protocols, which have different Hamiltonian endpoints, it is useful to introduce a measure of speed defined as $v=\|\Delta H\|/N$, where $\|\Delta H\|$ denotes the operator norm of the change in the system's Hamiltonian. Together with the expressions above, this is used to compute Fig. 2 in the main text that compares incoherent to coherent processes.

II. SUPPLEMENTARY NOTE 2: ERROR ANALYSIS

We discuss how statistical and systematic errors for the quantity $N\mathcal{Q}/\|\Delta H\|$ shown in Fig. 2 and 3 of the main text (with N being the number of subdivision and $\|\Delta H\|$ being the (operator) norm of the change in the qubit's Hamiltonian) are estimated.

A. Parameter extraction

Each measured value of the quantity $N\mathcal{Q}/\|\Delta H\|$ is estimated from $M = 8000$ independent runs, each consisting of N *two-point energy measurements* (TPM). Each TPM is comprised of two single-qubit readouts in the computational basis. The inverse effective temperature β is determined by the probability p of the qubit to be in $|0\rangle$ after the first measurement, according to Eq. (3) of the main text. With a binomial statistical error $\sigma^{(\mathrm{b})} = \sqrt{p(1-p)/(N\,M)}$, the statistical error of β is given by

$$\sigma_\beta = \frac{d\beta}{dp}|_p \sigma^{(\mathrm{b})}. \tag{18}$$

The probability of the result of the second measurement for each TPM being different from the first result is given by the flip probability p_f and can therefore be estimated from the overall frequency of flipped results. It is also subject to the same binomial statistical errors $\sigma_f^{(\mathrm{b})} = \sqrt{p_f(1-p_f)/(N\,M)}$.

B. Statistical errors of $\mathcal{Q}$

The measured values of the quantum correction to the FDR $\mathcal{Q}$ are computed from: β, the average work $\langle W\rangle$ and its variance Var(W) (see Eq. (5) of the main text). The resulting statistical error of $\mathcal{Q}$ is computed via bootstrapping, where for each parameter set $(N, \beta, \theta = \pi/(4N))$, 200 artificial data sets are generated, each consisting of NM statistically independent TPMs. This is done by randomly initializing the qubit in the "dark" state vector $|0\rangle$ with a probability p and then flipping it with a probability p_f. For each of the 200 data sets, the mean work, work variance and β are determined, from which a bootstrapping value of $\mathcal{Q}$ is computed. The bootstrapping statistical error $\sigma_{\mathcal{Q}}^{(BS)}$ of a given $\mathcal{Q}$ value is the sample standard deviation of the the 200 $\mathcal{Q}$ values obtained for each data set. Finally, $\sigma_{\mathcal{Q}}^{(BS)}$ includes the bootstrapping errors $\sigma_\beta^{(BS)}$ on the inverse temperature (through Eq. (9)), which are shown as horizontal error bars in Fig. (3) of the main text.

C. State preparation and measurement (SPAM) errors

Both state preparation and measurement readout of the qubit are error-prone. After the first measurement of each TPM, the qubit is reinitialized via optical pumping on the quadrupole transition and an optional π-pulse. The state preparation error for both these processes is very small $\lesssim 0.1\%$, and therefore it can be neglected. For each TPM, the state is re-prepared based on the result of the first measurement, and the true value of p is given by the dark event probability of the first measurement slot. The determination of β can be considered to be free of state preparation and measurement errors.

However, measurement errors have to be taken into account for the second measurement of each TPM, based on the conditional error probabilities $p_{b|0}$ ($p_{d|1}$) of incorrectly reading out qubit as "bright" ("dark") when it has actually been in $|0\rangle$ ($|1\rangle$). These conditional error probabilities are determined from separate calibration measurements, which merely consist of state preparation both in $|0\rangle$ and $|1\rangle$ and readout. Using at least 10000 shots for each calibration measurement, we determine readout error rates of about 0.4 ± 0.1 %.

The measurement error leads to modified probabilities to measure nonzero work events within each TPM protocol. In particular, Eq. (12) becomes

$$P'(w_j = +1) = (1 - p_{d|1})P(w_j = +1) + p_{b|0}P(w = 0), \tag{19}$$

$$P'(w_j = -1) = (1 - p_{b|0})P(w_j = -1) + p_{d|1}P(w_j = 0), \tag{20}$$

where $P(w_j = \pm 1, 0)$ are the probabilities defined in Eq. (12). Eq. (19) gives rise to spurious values of the correction to the classical FDR we we call $\mathcal{Q}_{\text{SPAM}}$. Specifically, in order to single out the effect of such SPAM errors, we compute $\mathcal{Q}_{\text{SPAM}}$ when coherent drive on the qubit's Hamiltonian is performed (i.e., $\theta = 0$) and only TPM readouts are carried out according to Eq. (19). This quantity provides the worst-case estimation of a spurious FDR correction, which reads

$$\mathcal{Q}_{\text{SPAM}} = N\left[\frac{\beta}{2}\left[-(p\,p_{d|1} - (1-p)p_{b|0})^2 - pp_{b|0} + p\,p_{d|1} + p_{b|0}\right] + \left[p\,p_{d|1} - (1-p)p_{b|0}\right]\right]. \tag{21}$$

Eq. (21) clearly shows that this quantity linearly grows with the number of subdivisions N, thus becoming the predominant contribution in the slow driving regime $N \gg 1$.

D. Parameter drifts

The parameters β and θ are determined by laser intensities, which are subject to drifts over the course of the data acquisition. For each value of $\mathcal{Q}$, the parameters are estimated from MN runs. In order to analyze the impact of such potential drifts, these runs are partitioned into $(MN)/K$ bins of size K each, and the probabilities p (from which β is computed) and p_f (from which θ is computed) are calculated for each bin. Then, the standard deviation among the various p and p_f is computed and compared to the standard deviation expected from a binomial distribution, as explained in Subsec. B.1. For all data sets, no significant excess spread is observed, i.e., $\delta p, \delta p_f < 0.01$ throughout each estimation of $\mathcal{Q}$. We conclude that drift errors are therefore negligible.

III. SUPPLEMENTARY NOTE 3: EXPERIMENTAL IMPLEMENTATION DETAILS

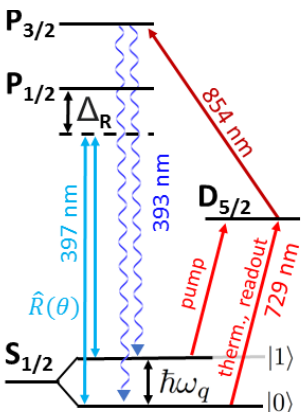

FIG. Supplementary 1. Level scheme of the employed $^{40}Ca^{+}$ qubit, showing all relevant states, transitions and wavelengths.

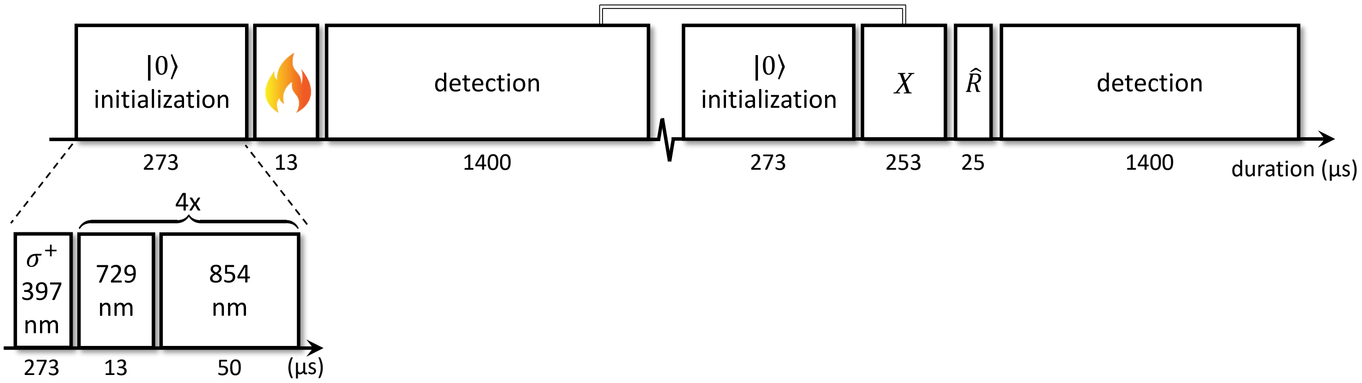

FIG. Supplementary 2. Timing sequence diagram for one cycle of the experiment depicted in Fig. 1a) of the main text. The inset shows the laser pulses for qubit initialization, see Fig. 1b) of the main text for the driven transitions. A flame depicts the thermalization step. Detection using a 729 and 397 nm laser determines whether to execute an X gate on a newly initialized qubit. A small coherent rotation $\hat{R}$ is performed by two 397 nm beams driving a Raman transition on the $S_{1/2}$ ground state, followed by a final detection.

A timing diagram detailing the laser driven qubit operations realizing the steps of the protocol is shown in Fig. Supplementary 2. In the following sections, we discuss the state preparation, the readout and the pulse area calibration in detail.

A. State preparation

Each step of the work measurement protocol begins with initializing the qubit in the logical basis state vector $|0\rangle$ with two-stage optical pumping. First, we drive the trapped $^{40}Ca^{+}$ ion resonantly on the $4^2S_{1/2} \leftrightarrow 4^2P_{1/2}$ transition using a circularly polarized driving field, such that the selection rules lead to depletion of $|1\rangle$ and population of $|0\rangle$. The second pumping stage consists of repeated selective depletion of $|1\rangle$ via a series of approximate π-pulses driving the narrow $4^2S_{1/2} \leftrightarrow 3^2D_{5/2}$ electric quadrupole transition at about 729 nm, each pulse followed by depletion of the meta-stable state by driving the $3^2D_{5/2} \leftrightarrow 4^2P_{3/2}$ dipole transition near 854 nm. The natural linewidth of about 1 Hz of the electric quadrupole transition leads to a resolved Zeeman substructure, such that the level $|1\rangle$ is depleted in a frequency-selective manner. Using this two-step scheme, a state preparation fidelity of better than 99% is obtained.

The preparation of any desired Gibbs state (Eq. (4) in the main manuscript) is accomplished by selective population transfer on the electric quadrupole transition, in conjunction with the first measurement of the TPM: First, a fixed amount of population is transferred from $|0\rangle$ to the metastable $^2D_{5/2}$ state. The first readout of the TPM projects the qubit either in $|0\rangle$ or $|1\rangle$ according to the occupation probabilities. The probability to detect the qubit in $|1\rangle$ ('bright') corresponds to the Boltzmann weight pertaining to state $|1\rangle$ (Eq. (5) in the main manuscript). Note that the protocol does not require the precise setting of the values of β: We tune the pulse duration of the 729 nm transfer to roughly accomplish a desired Boltzmann factor, and the infer the actual value of β from the respective measurement data, by evaluating only the results from the first TPM slot, which then allows for estimating β via Eq. (5) (of the main manuscript).

B. Qubit readout

Readout of the qubit in the logical basis vectors $|0\rangle$, $|1\rangle$ is accomplished by a two-step process: First, similarly to the second stage of the optical pumping, population is selectively transferred from $|0\rangle$ to the metastable $^2D_{5/2}$ state. Here, we as we require robust and complete population transfer, we employ frequency-chirped laser pulses with Gaussian intensity envelope to realize rapid adiabatic passage (RAP). This allows for readout fidelities of about 99% throughout long data acquisition times. In the second step, the ion is illuminated near 397 nm, resonantly driving the $4^2S_{1/2} \leftrightarrow 4^2P_{1/2}$ transition. This leads to the detection of resonance fluorescence only for the population which has *not* been transferred to the metastable state in the previous stage. The resonance fluorescence is collected with a lens and detected by a photomultiplier tube. If the number of detected photons within the readout duration exceeds predefined threshold, the ion has been detected in $|1\rangle$ ('bright'), otherwise it is detected in $|0\rangle$ ('dark').

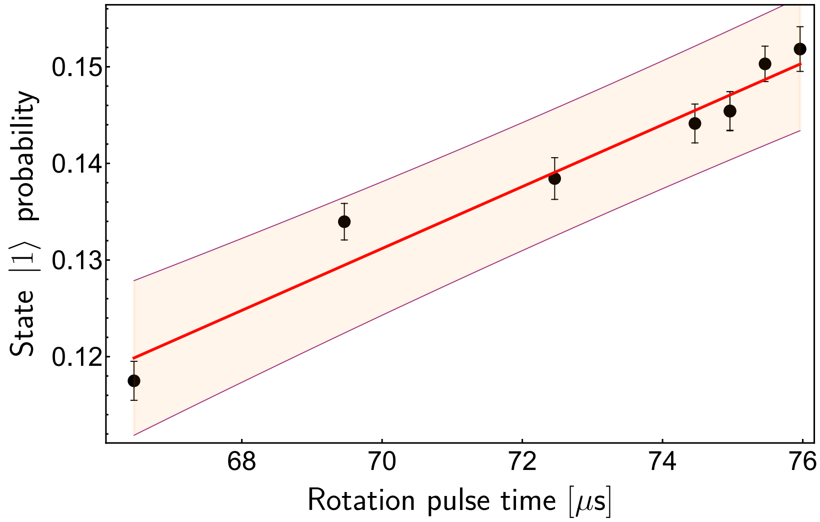

FIG. Supplementary 3. Small-area rotation pulse calibration for $N = 2$. The error bars of the data points are calculated with non-parametric bootstrapping, the red line represents the linear regression fit, and the purple lines, together with the shaded area, represent the prediction confidence interval at 95%.

This readout operation completely depolarized the qubit. In order to realize a viable TPM, the qubit has to be reset according to the result of the first measurement. This is accomplished by another initialization in $|0\rangle$ as described above, followed by a Pauli $\hat{X}$ gate (spin flip) realized on the stimulated Raman transition with a rotation angle of π, which is *only* carried out if the

previous measurement result has been $|1\rangle$. This way, the qubit state is the same as for an ideal projective measurement, which also holds true for the prepared Gibbs ensemble state.

C. Pulse area calibration

The rotation angle $\Delta\theta$ is calibrated experimentally by separate measurements, which consist of preparing the qubit in state $|0\rangle$, then illuminating the ions with the off-resonant beams near 397 nm, i.e., resonantly driving the stimulated Raman transition with a pulse of duration t_p, and finally performing a measurement. This experiment is repeated a few thousand times for different values of t_p, while keeping the intensity, and thus the effective Rabi frequency Ω constant. The probability of reading out "bright" (corresponding to $|1\rangle$) is given by $P_{\text{bright}} = \sin^2(\Omega t_p)/2)$ [2]. For modestly large values of N (and correspondingly small values of $\Delta\theta$), as P_{bright} is not a linear function t_p, we chose closely-spaced values of t_p such that P_{bright} can be linearized. We can then determine the required t_p by linear regression. This calibration measurement is carried out for each value of N, with different sample pulse times t_p centered near the estimated target pulse time. From the linear regression results, a best estimate of the required pulse time realizing $\Delta\theta = \pi/(2N)$ is obtained, including an estimation of its accuracy. For increasing values of N and correspondingly decreasing values of $\Delta\theta$ and P_{bright}, an increasing fraction of the observed 'bright' events arises from measurement errors. This leads to a systematic underestimation of the required pulse time, which explains why the observed Q values systematically fall short with respect to the ideal values, for the case of low temperatures.

[1] M. Scandi, H. J. D. Miller, J. Anders, and M. Perarnau-Llobet, Phys. Rev. Research **2**, 023377 (2020).
[2] C. J. Foot, *Atomic physics* (Oxford University Press, 2005).
